# A Rare Case of Adnexal Pregnancy After Bilateral Tubal Clamping

**DOI:** 10.7759/cureus.43284

**Published:** 2023-08-10

**Authors:** Aishwarya Gupta, Sandhya Pajai, Deepti Shrivastava, Aditi Singh Thakur, Muneeba Shaikh

**Affiliations:** 1 Department of Obstetrics and Gynecology, Jawaharlal Nehru Medical College, Datta Meghe Institute of Higher Education and Research, Wardha, IND

**Keywords:** in vitro fertilization (ivf), medical managment, repeat ectopic, tubal ligation, tubal ectopic pregnancy

## Abstract

An adnexal pregnancy after tubal clamping is a very rare entity. Very few such cases have been reported in the past. Here, we discuss a case of such an occurrence. A 35-year-old female with third gravida and a history of two abortions with secondary infertility conceived via in vitro fertilization was admitted to the ward for observation with a history of amenorrhea of one and a half months and a known case of hypothyroidism. The ultrasonography showed left ectopic adnexal pregnancy that was managed conservatively. With a previous history of left ectopic pregnancy, the patient was managed with exploratory laparotomy with left partial salpingectomy.

This case is used to illustrate the need to gather a complete medical history and take ectopic pregnancy into account in women who are of reproductive age and have a history of ectopic pregnancies.

## Introduction

Ectopic pregnancy is a major cause of morbidity and/or death in the first three months of pregnancy, and the rate of occurrence considerably rises after in vitro fertilization (IVF) or embryo transfer [1]. Rarely, an ectopic pregnancy can also develop in the ovaries, the abdominal cavity, or even the cervix, which is the lower portion of the uterus connected to the vagina.

The Centers for Disease Control and Prevention estimate that ectopic pregnancy comprises approximately 2% of all documented pregnancies. Up to 18% of women who attend the emergency room in the first trimester with abdominal pain or vaginal bleeding or both are thought to be carrying ectopic pregnancies. With more than 90% of instances occurring in the fallopian tube, ectopic implantation occurs there most frequently [2]. However, because of delayed identification and treatment, 1% of implantation can occur in the cervix, 1-3% in the ovary, and 1-3% in previous cesarean scars, frequently leading to increased morbidity [3]. Ultrasonography, especially transvaginal ultrasound, and quantification of the beta-human chorionic gonadotropin (beta-hCG) are the cornerstone of diagnosis. The basis for the ultrasound diagnosis is the visualization of an ectopic mass rather than the difficulty to envision a pregnancy within the uterus [4].

The idea that there is a beta-hCG value at which the ultrasound characteristics of a typical intrauterine gestation should be seen is known as the *discriminatory level*. An ultrasound with no detectable gestational sac and a beta-hCG result over the discriminating level indicates an unviable pregnancy (an early pregnancy loss or an ectopic pregnancy) [1]. It is possible to treat proven ectopic pregnancies conservatively with intravenous or intramuscular methotrexate, operatively with salpingostomy or salpingectomy, and, in a few cases, expectantly. Immediate transfer for operative management is crucial if a patient presents with hemodynamic instability or peritoneal signs, exhibits high baseline beta-hCG levels, or has fetal cardiac activity detected outside the uterus via ultrasonography. It is imperative to take prompt action when conservative or expectant management is not an option or has failed [5].

## Case presentation

A 35-year-old married multiparas woman with two previous abortions who was eager to conceive for seven years was diagnosed with secondary infertility and presented to the hospital for embryo transfer, which was done on June 11. Following this, she was admitted for observation. On June 22, that is, 15 days after the embryo transfer, the first sample of beta-hCG showed a value of 200 mIU/mL, which was suggestive of pregnancy f/b. The second sample sent on June 24 showed a value of 746 mIU/mL, which was suggestive of doubling and confirmed pregnancy. Her urine pregnancy test (UPT) came back positive on July 7. The patient also had a history of hypothyroidism for four years on tablet Thyrox 25 µg OD.

The patient had a history of two failed in-utero inseminations and one ovum pickup done in 2020. She had a history of one failed IVF conception in 2021. She underwent diagnostic hysteroscopy in 2021, which was suggestive of a normal uterine cavity with bilateral Ostia. She also had a history of laparoscopic ovarian drilling in 2021, which was suggestive of adhesions with left hydrosalpinx.

In the first pregnancy, she conceived naturally. It was a ruptured ectopic tubal pregnancy with a hemodynamically compromised state, for which the patient underwent exploratory laparotomy with left partial salpingectomy along with clipping for the right fallopian tube (in 2015).

In her second pregnancy, she conceived via IVF (embryo transfer was done). UPT was positive, but on ultrasonography, absent cardiac activity was noted. Subsequently, medical termination of pregnancy was done, and no suction evacuation was required.

In the present pregnancy, she conceived via IVF. On July 7 (day 0), the patient complained of pain in the abdomen in the left iliac region that was dull aching, non-radiating, associated with per vaginum spotting, and not associated with the passage of clots.

The patient appeared stable and alert, with a pulse rate of 98 beats per minute and a blood pressure of 110/80 mmHg. During the physical examination, she experienced the most discomfort in the lower abdomen, specifically in the left iliac fossa. A pelvic examination revealed a normal-sized uterus with a firm cervix and tenderness in the left fornix. Additionally, cervical motion tenderness was observed. Complete hemogram results were found to be within normal ranges.

On day one, beta-hCG showed a value of 12,139 mIU/mL, which was confirmatory of the ultrasonography finding of ectopic pregnancy. As the patient was hemodynamically stable, she was managed medically with serial readings of beta-hCG and ultrasonography.

She was managed conservatively by administering tablet mifepristone 200 mg on day one along with injection methotrexate 1 mg/kg intramuscularly on days one, three, five, and seven for a total of four doses, alternating with injection folinic acid 0.1 mg/kg intramuscularly on days two, four, six, and eight. On day nine, ultrasonography was done along with serum beta-hCG, with a value of 3,337.1 mIU/mL.

The patient was discharged on tablet Autrin once a day for one month and syrup b-complex (two teaspoons) twice a day for one month, and was advised weekly follow-up for serum beta-hCG testing. She came back on July 25 with a beta-hCG value of 1,038.4 mIU/mL and again on August 3 with a value of 14.43 mIU/mL.

As the size of the heterogenous adnexal mass decreased and there was no vascularity on ultrasonography, along with decreasing beta-hCG levels, it was inferred that there was no further need for surgical intervention. All beta-hCG values along with the serial ultrasonography findings are summarized in Table 1.

**Table 1:** Summary of serial beta-human chorionic gonadotropin (beta-hCG) values and ultrasonography findings. MSD: mean sac diameter

Date	Beta-hCG value	Ultrasonography findings
8/7/22	12,139 mIU/mL	A cystic structure with surrounding decidual reaction was noted between the left ovary and uterus measuring approximately 1.5 × 1.3 cm - p/o left adnexal ectopic pregnancy with no evidence of yolk sac, fetal pole, or cardiac activity
10/7/22	12,573 mIU/mL	A gestational sac measuring 7 mm corresponding to five weeks and three days with surrounding decidual reaction. No yolk sac and fetal pole/cardiac activity were noted
12/7/22	12,032 mIU/mL	An irregular gestational sac measuring 6 mm. The lesion (gestational sac and decidual reaction) measured 1.9 × 1.8 × 1.7 cm. There was no yolk sac or fetal pole/cardiac activity. There was minimal free fluid in the pouch of Douglas with internal echoes
14/7/22	4,396.2 mIU/mL	A heterogeneously hyperechoic lesion measuring 1.9 × 1.8 × 1.6 cm in the left adnexa. The gestational sac was not visualized in the present scan. Free fluid in the pouch of Douglas decreased compared to the previous scan
16/7/22	3,337.1 mIU/mL	The present scan revealed a heterogenous lesion measuring 1.9 × 1.8 × 1.7cm in the left adnexa. gestational sac/fetal pole, not visualized in the present scan, minimal free fluid in the pod (no significant change compared to the previous scan)
25/7/22	1,038.4 mIU/mL	No scan was done
3/8/22	14.43 mIU/mL	No scan was done

The patient was also counseled for follow-up for hysterosalpingography to rule out utero-peritoneal fistula; however, the patient was not willing to undergo further follow-up or treatment. The couple received advice on comprehensive preconception counseling, planned pregnancy, and the usage of a barrier form of contraception for six months.

## Discussion

Ectopic pregnancies occur about one in 100 times. If the patient had continued prior contraception, the probability of ectopic pregnancy was 12.5%, which is lower than what is typically seen in healthy women [6]. While some tubal ectopic pregnancy cases spontaneously resolve, some cases persist and run the risk of rupturing the tube. An ectopic pregnancy occurs when the ova are implanted outside the uterus after fertilization. It usually implants inside a fallopian tube, which transports ova from the ovaries to the uterus. Tubal pregnancy is another term for this type of ectopic pregnancy [7].

**Figure 1:**
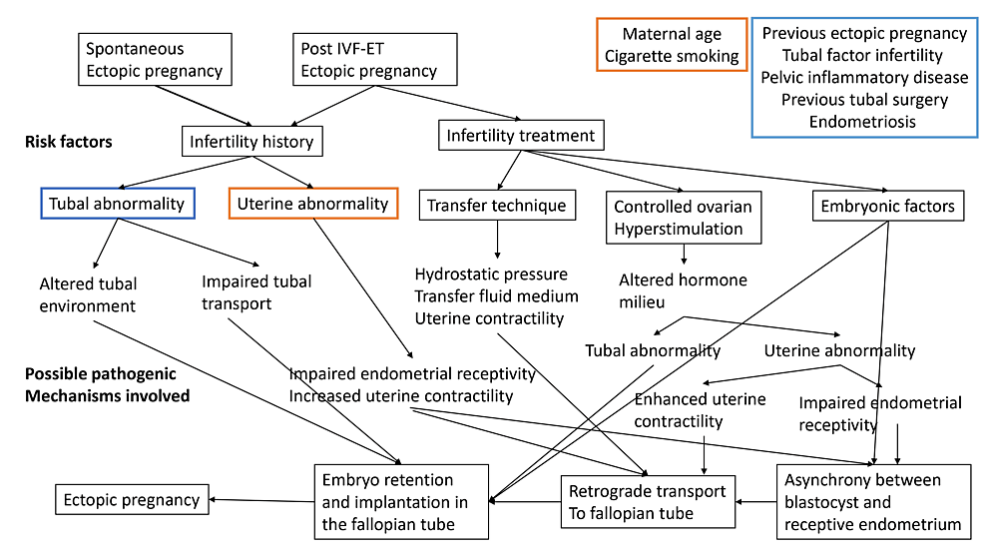
Pathogenic mechanisms. Potential processes, in connection to known risk factors, that contribute to the pathophysiology of tubal pregnancy following spontaneous and in vitro fertilization conception. Figure reproduced from Refaat et al. [8] published under the CC BY license.

As one of them may simultaneously be a cause and a result of the other, the relationship between infertility and ectopic pregnancy is complicated. Following reproductive therapy, there is a higher chance of ectopic pregnancy, which may be caused by the medication’s side effects or a pre-existing condition [8]. As both naturally occurring tubal pregnancies and infertility treatment tubal pregnancies share comparable tubal risk factors, any type of tubal damage probably plays a substantial role in the disease process of both. Figure 1 summarizes the potential pathogenic processes linked to risk factors for ectopic pregnancy after natural or spontaneous conception or conception via assisted techniques [8].

Women with fallopian tube damage from pelvic diseases, pelvic operations, or a previous ectopic pregnancy, as well as smokers, are more likely to experience an ectopic pregnancy. Although the use of intrauterine devices does not increase the chance of ectopic pregnancy in absolute terms, it does increase the likelihood that the pregnancy will be extrauterine.

The risk of recurrence for tubal ectopia is 10% after a salpingectomy, 15% after a salpingotomy, and varies from 10.2% to 18.7% after receiving medical attention. According to reports, the prevalence of ectopic pregnancy increased from 0.5% to 2% over 30 years [7]. Early detection of ectopic pregnancy requires a strong index of suspicion. The available treatments for ectopic pregnancy are expectant, conservative, medical, and operative management [8]. Patients who have low or decreasing beta-hCG levels should be managed expectantly. Patients who have hemodynamically unstable circumstances, heterotopic pregnancy, tubal rupture, and unsuccessful medical treatment should consider surgery [5].

Medical care may be effective in some individuals who have elevated pretreatment beta-HCG levels, and the high beta-HCG level itself is not a reason to avoid it [9]. In this case, given the patient’s stable hemodynamic status with decreasing beta-hCG levels, medical management was the treatment of choice.

## Conclusions

Early detection of ectopic pregnancy depends on a strong index of suspicion. The available treatments for ectopic pregnancy include expectant management, surgical management, or medical management. In cases of prior ectopic pregnancies, the frequency of ectopic pregnancies is significant. IVF techniques also pose an increased risk of ectopic pregnancy. In this case, an infectious tubal illness may be the cause of a rare occurrence of subsequent ectopic pregnancy following unilateral salpingectomy. As the patient’s hemodynamics were stable, medical management was decided.
